# Whole blood methylome-derived features to discriminate endocrine hypertension

**DOI:** 10.1186/s13148-022-01347-y

**Published:** 2022-11-03

**Authors:** Roberta Armignacco, Parminder S. Reel, Smarti Reel, Anne Jouinot, Amandine Septier, Cassandra Gaspar, Karine Perlemoine, Casper K. Larsen, Lucas Bouys, Leah Braun, Anna Riester, Matthias Kroiss, Fidéline Bonnet-Serrano, Laurence Amar, Anne Blanchard, Anne-Paule Gimenez-Roqueplo, Aleksander Prejbisz, Andrzej Januszewicz, Piotr Dobrowolski, Eleanor Davies, Scott M. MacKenzie, Gian Paolo Rossi, Livia Lenzini, Filippo Ceccato, Carla Scaroni, Paolo Mulatero, Tracy A. Williams, Alessio Pecori, Silvia Monticone, Felix Beuschlein, Martin Reincke, Maria-Christina Zennaro, Jérôme Bertherat, Emily Jefferson, Guillaume Assié

**Affiliations:** 1 Université Paris Cité, CNRS, INSERM, Institut Cochin, F-75014 Paris, France; 2 Division of Population Health and Genomics, School of Medicine, University of Dundee, Dundee, DD2 4BF UK; 3 Institut Curie, INSERM U900, MINES ParisTech, PSL-Research University, CBIO-Centre for Computational Biology, Paris, France; 4 Sorbonne Université, INSERM, UMS Production et Analyse de données en Sciences de la vie et en Santé, PASS, Plateforme Post-génomique de la Pitié-Salpêtrière, P3S, 75013 Paris, France; 5 Université Paris Cité, Inserm, PARCC, F-75015 Paris, France; 6 Medizinische Klinik und Poliklinik IV, Klinikum der Universität, Ludwig-Maximilians-Universität München, Munich, Germany; 7 Service d’Hormonologie, AP-HP, Hôpital Cochin, F-75014 Paris, France; 8 Unité Hypertension Artérielle, AP-HP, Hôpital Européen Georges Pompidou, 75015 Paris, France; 9 Centre d’Investigations Cliniques 9201, AP-HP, Hôpital Européen Georges Pompidou, F-75015 Paris, France; 10 Département de Médecine Génomique des Tumeurs et des Cancers, Hôpital Européen Georges Pompidou, F-75015 Paris, France; 11 Department of Hypertension, Institute of Cardiology, Warsaw, Poland; 12 BHF Glasgow Cardiovascular Research Centre, Institute of Cardiovascular and Medical Sciences, University of Glasgow, Glasgow, G12 8TA UK; 13 Department of Medicine-DIMED, Emergency and Hypertension Unit, University of Padova, University Hospital, Padua, Italy; 14 UOC Endocrinologia, Dipartimento di Medicina DIMED, Azienda Ospedaliera-Università di Padova, Padua, Italy; 15 Division of Internal Medicine and Hypertension Unit, Department of Medical Sciences, University of Torino, Turin, Italy; 16 Klinikfür Endokrinologie, Diabetologie Und Klinische Ernährung, UniversitätsSpital Zürich (USZ) and Universität Zürich (UZH), Raemistrasse 100, 8091 Zurich, Switzerland; 17 Service de Génétique, AP-HP, Hôpital Européen Georges Pompidou, F-75015 Paris, France; 18 Service d’Endocrinologie, Center for Rare Adrenal Diseases, AP-HP, Hôpital Cochin, F-75014 Paris, France; 19 Institute of Health and Wellbeing, University of Glasgow, Glasgow, G12 8RZ UK

**Keywords:** Endocrine hypertension, Whole blood methylome, Circulating biomarker

## Abstract

**Background:**

Arterial hypertension represents a worldwide health burden and a major risk factor for cardiovascular morbidity and mortality. Hypertension can be primary (primary hypertension, PHT), or secondary to endocrine disorders (endocrine hypertension, EHT), such as Cushing's syndrome (CS), primary aldosteronism (PA), and pheochromocytoma/paraganglioma (PPGL). Diagnosis of EHT is currently based on hormone assays. Efficient detection remains challenging, but is crucial to properly orientate patients for diagnostic confirmation and specific treatment. More accurate biomarkers would help in the diagnostic pathway. We hypothesized that each type of endocrine hypertension could be associated with a specific blood DNA methylation signature, which could be used for disease discrimination. To identify such markers, we aimed at exploring the methylome profiles in a cohort of 255 patients with hypertension, either PHT (*n* = 42) or EHT (*n* = 213), and at identifying specific discriminating signatures using machine learning approaches.

**Results:**

Unsupervised classification of samples showed discrimination of PHT from EHT. CS patients clustered separately from all other patients, whereas PA and PPGL showed an overall overlap. Global methylation was decreased in the CS group compared to PHT. Supervised comparison with PHT identified differentially methylated CpG sites for each type of endocrine hypertension, showing a diffuse genomic location. Among the most differentially methylated genes, *FKBP5* was identified in the CS group. Using four different machine learning methods—Lasso (Least Absolute Shrinkage and Selection Operator), Logistic Regression, Random Forest, and Support Vector Machine—predictive models for each type of endocrine hypertension were built on training cohorts (80% of samples for each hypertension type) and estimated on validation cohorts (20% of samples for each hypertension type). Balanced accuracies ranged from 0.55 to 0.74 for predicting EHT, 0.85 to 0.95 for predicting CS, 0.66 to 0.88 for predicting PA, and 0.70 to 0.83 for predicting PPGL.

**Conclusions:**

The blood DNA methylome can discriminate endocrine hypertension, with methylation signatures for each type of endocrine disorder.

**Supplementary Information:**

The online version contains supplementary material available at 10.1186/s13148-022-01347-y.

## Background

Arterial hypertension affects over a billion people worldwide, with an estimated overall prevalence in adults of around 30–45% [1, 2]. This is one of the major risk factors for multiple cardiovascular and renal disorders and represents one of the most preventable causes of morbidity and mortality. Around 5 to 10% of arterial hypertension cases are estimated to have secondary causes, most commonly due to parenchymal renal disease, renovascular hypertension, obstructive sleep apnea, or endocrine diseases, such as Cushing’s syndrome (CS), primary aldosteronism (PA), and pheochromocytoma/paraganglioma (PPGL) [3, 4]. Diagnostic screening for secondary hypertension is complex and expensive [5] and generally restricted to patients with clinical signs, including hypertension in young adults, sudden worsening of blood pressure in normotensive subjects, and drug-resistant hypertension, among others [3, 4]. However, the prevalence of secondary causes of hypertension is often underestimated and many cases remain unrecognized [6–8]. Early detection of endocrine forms of hypertension is crucial to control high blood pressure and prevent hypertension-mediated organ damage and related cardiovascular complications, as well as achieve effective long-term treatment [9]. Moreover, hormone excess may increase individual risk of other consequences beyond hypertension, particularly in the case of CS or PA [10–12].

Currently, the diagnosis of endocrine hypertension (EHT) relies on hormonal evaluations, with specific diagnostic algorithms for each type of endocrine disorder. Hormone assays present some limitations, including (i) common borderline values, especially in mild forms of over-secretion, where the diagnosis may be missed or wrongly called; (ii) lack of direct estimation of the individual risk to hormone exposure, despite the important inter-individual variability of hormone excess consequences; (iii) recommendation for multiple hormonal tests, with screening and confirmatory strategies still debated, and varying from one center to the other [13]. New biomarkers could potentially allow to directly measure tissue exposure to hormone excess, with the potential improvement of diagnostic accuracy and prediction of individual susceptibility to different consequences.

Circulating biomarkers in the blood have the advantage of being non-invasive when utilized. Leukocyte DNA methylation is particularly convenient as a biomarker, being a chemically stable yet dynamic biological hallmark, with a key role in epigenetic regulation in both health and disease [14]. Recent epigenome-wide association studies exploring leukocyte DNA methylation in hypertensive patients versus normotensive controls identified different loci associated with blood pressure regulation [15–17]. However, it is not yet established whether blood methylation profiles could also discriminate patients with EHT from those with primary hypertension (PHT). Indeed, hormone excess may impact peripheral tissues at the epigenetic level, measurable as hormone-specific methylome signatures. This hypothesis is supported by a recent work, in which we were able to identify a blood methylome signature of CS [18].

In the present study, we explored leukocyte methylation profiles in hypertensive patients. Specifically, we analyzed whole blood methylome signatures in patients with PHT or EHT related to CS, PA, or PPGL.

## Results

### Patients

Blood samples were collected from 255 patients. Patients had been diagnosed either with primary hypertension (PHT, *n* = 42), or endocrine forms of hypertension (EHT, *n* = 213), either related to the presence of CS (*n* = 57), PA (*n* = 101), or PPGL (*n* = 55). Each group, except PA, showed a predominance of female patients, and the mean age was lower in patients with EHT than in patients with PHT (*p* < 0.01; Table 1).

**Table 1:** Sex and age distribution in the different hypertension types

Diagnosis	n Total	Sex		*p* value*	Age	*p* value**
		n Female	n Male		Mean ± sd	
PHT	42	25 (60%)	17 (40%)		55.5 ± 11.5	
EHT	213	119 (60%)	94 (40%)	ns	48.5 ± 11.6	< 0.01
*EHT*						
CS	57	40 (70%)	17 (30%)	ns	48.1 ± 11.7	< 0.01
PA	101	43 (43%)	58 (57%)	ns	47.8 ± 9.4	< 0.01
PPGL	55	36 (65%)	19 (35%)	ns	50.1 ± 14.8	< 0.05

Sex and age distribution are provided for each type of hypertension, and compared to primary hypertension (PHT); *ns* not significant

*Chi-square test

**Student’s t-test

### Whole blood DNA methylome profile in hypertension

**Fig. 1:**
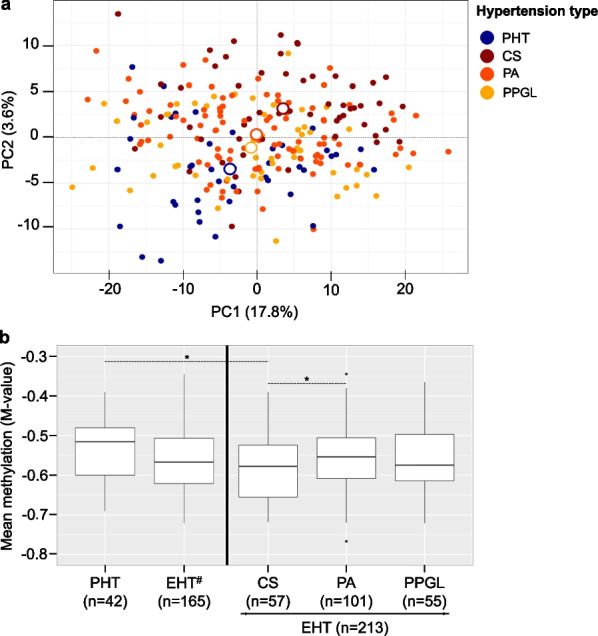
Global structure of blood DNA methylation profile in different hypertension types. **a** Sample projections based on the first two principle components (PC1, PC2) of unsupervised PCA performed on the whole dataset (*n* = 731,635 CpG sites, *n* = 255 samples). The center of each group is indicated by the larger white circles. **b** Boxplot of the most variable CpG sites (*n* = 48,452 with M-Value SD > 0.4) in the four hypertension types. ^#^CS, PA and PPGL were aggregated into a single EHT type, with random sampling of 55 samples from each group. *Student’s t-test p value < 0.05

Whole-genome blood DNA methylome was determined for the 255 samples, with 731,635 informative CpG sites in all samples. Unsupervised principal component analysis showed a distribution of samples with separation depending on the type of hypertension (Fig. 1a). The main components of variability were associated with the type of hypertension, but also white blood cell count variation and, to a lower extent, with age and sex (Additional file 1: Fig. S1).

The mean methylation level of the most variable CpG sites among all samples (*n* = 48,452) was not significantly different between the different types of hypertension, except in the group of CS patients, where methylation was decreased (Fig. 1b).

### The methylome signature of endocrine hypertension

**Fig. 2:**
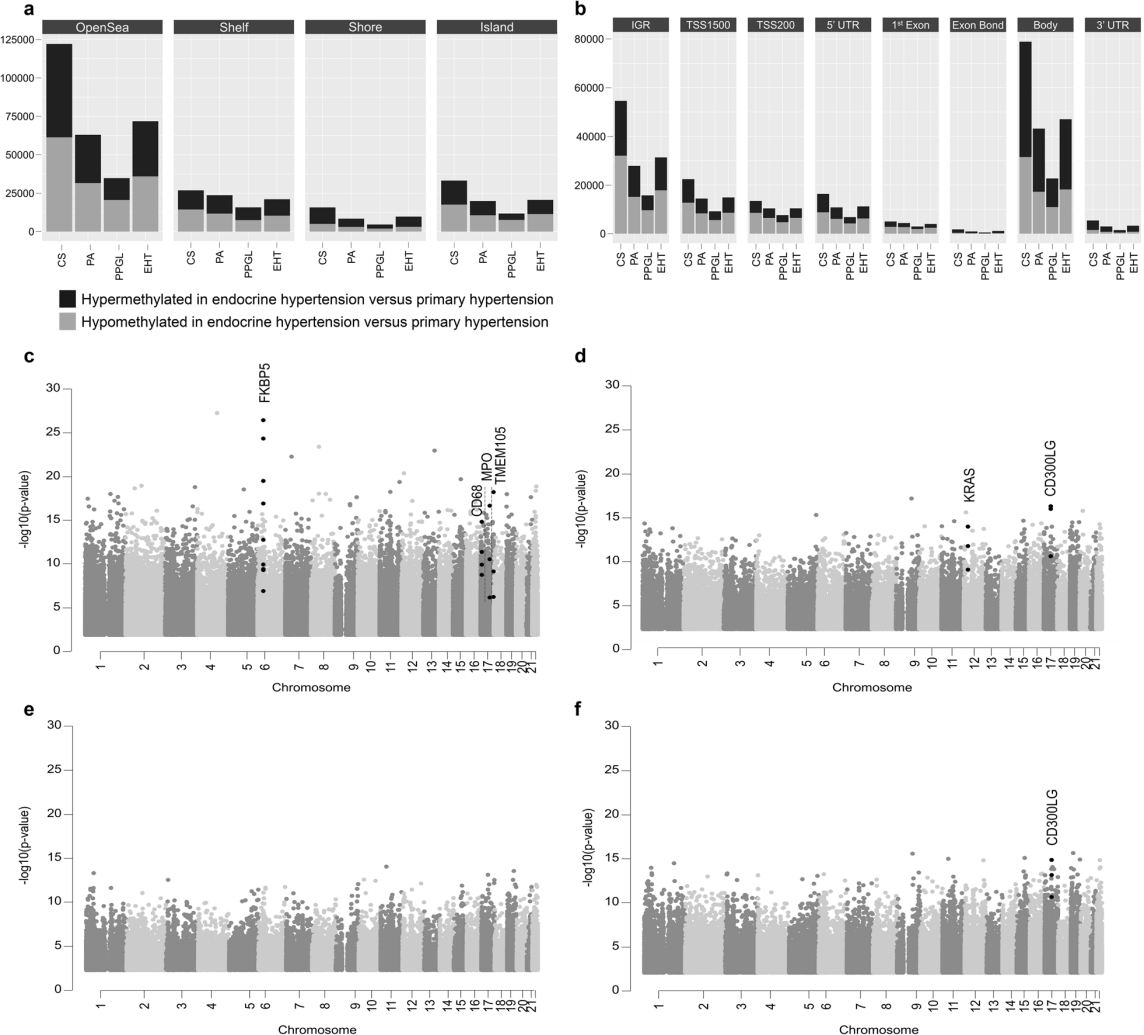
Characteristics of differentially methylated CpG sites in endocrine hypertension. **a** Distribution of differentially methylated CpG sites related to CpG genomic density. **b** Distribution of differentially methylated CpG sites related to genes structure. **c**–**f** Manhattan plots representing the genomic distribution of differentially methylated CpG sites for each type of endocrine hypertension, in the comparisons CS vs. PHT (**c**), PA vs. PHT (**d**), PPGL vs. PHT (**e**), EHT vs. PHT (**f**). The most differentially methylated genes (mean –log10(*p* value) > 11, *Limma*) are highlighted in black

Comparing the 731,635 informative CpG sites of each type of EHT to PHT, differentially methylated CpG sites were identified in EHT (*n* = 123,288), CS (*n* = 197,897), PA (*n* = 114,988), and PPGL (*n* = 66,976) versus PHT, respectively (Additional file 2: Table S1). Differentially methylated CpG sites were distributed all over the genome (Fig. 2c–f), and the proportion of hypo- and hyper-methylated CpG sites was not strongly related to CpG genomic density (Fig. 2a), nor to CpG proximity to genes (Fig. 2b). Gene set enrichment analysis of genes associated with the differentially methylated CpG sites in the four comparisons revealed enrichment in pathways related to blood pressure regulation mechanisms and hypertension, including MAPK, Rap1, phospholipase D and calcium signaling pathways [19–23] (FDR < 0.001 in all comparisons; Additional file 3: Table S2).

Beyond the analysis of individual CpG sites, a specific analysis of differentially methylated genes was performed (Table 2 and Additional file 4: Table S3), highlighting the implication of specific genes for each type of EHT (Fig. 2c–f). The most significantly differentially methylated genes were *FKBP5* in CS and *CD300LG* in PA.

**Table 2:** Top 15 genes associated with the most differentially methylated CpG sites for each endocrine hypertension type

*CS vs. PHT*			*PPGL vs. PHT*			*PA vs. PHT*			*EHT vs. PHT*		
Gene	− log10 *p* value	logFC	Gene	− log10 *p* value	logFC	Gene	− log10 *p* value	logFC	Gene	− log10 *p* value	logFC
FKBP5	15.05	− 0.40	MUTYH	9.46	− 0.19	CD300LG	14.10	− 0.29	CD300LG	12.90	− 0.27
CD68	11.21	− 0.34	ELAVL1	9.43	− 0.28	KRAS	11.41	− 0.31	ZNF283	10.18	− 0.25
TMEM105	11.19	− 0.14	NLRC3	9.10	− 0.26	ZNF689	9.85	− 0.22	PTPN6	9.47	− 0.23
MPO	11.11	− 0.31	RASGEF1C	9.05	− 0.15	C16orf65	9.63	− 0.25	CCDC33	9.43	− 0.20
PDE6A	10.91	− 0.31	STAT6	8.89	− 0.26	C22orf32	9.42	− 0.36	STAT6	9.20	− 0.24
KDM4A	10.67	0.08	CD300LG	8.86	− 0.24	MFNG	9.33	− 0.26	MUTYH	9.20	− 0.19
PCDH24	10.63	− 0.05	CD44	8.69	− 0.23	GRB10	9.21	0.10	PTPRE	9.17	− 0.24
SLC1A5	10.60	− 0.41	MFNG	8.68	− 0.28	PLEKHA2	9.18	− 0.24	TCN2	9.13	− 0.13
GRK6	10.57	− 0.16	SLC35B1	8.60	− 0.24	SLC1A5	9.02	− 0.34	ARHGAP8	9.13	− 0.20
NAA50	10.43	− 0.32	PTPN6	8.58	− 0.25	GRHPR	8.98	− 0.27	GHDC	9.09	− 0.11
TTC22	10.40	0.10	C16orf65	8.51	− 0.26	CCRL2	8.96	− 0.27	SLC2A1	9.09	− 0.24
DIP2A	10.28	0.20	PADI4	8.31	− 0.26	FOXO1	8.91	− 0.36	SH2D3C	9.07	− 0.25
ADGRE5	10.25	− 0.31	FGD3	8.24	− 0.25	CACNA1G	8.90	− 0.41	NLRC3	9.00	− 0.23
MIR6840	10.25	0.19	TLCD1	8.21	0.30	CHD9	8.90	− 0.22	ELAVL1	8.99	− 0.13
TCEA2	10.13	0.01	DMKN	8.09	− 0.26	TIPARP− AS1	8.87	− 0.40	CD93	8.95	− 0.21

Group comparisons were performed by using *Limma* package, with Benjamin-Hochberg adjustment for multiple testing

### Prediction of endocrine hypertension

**Fig. 3:**
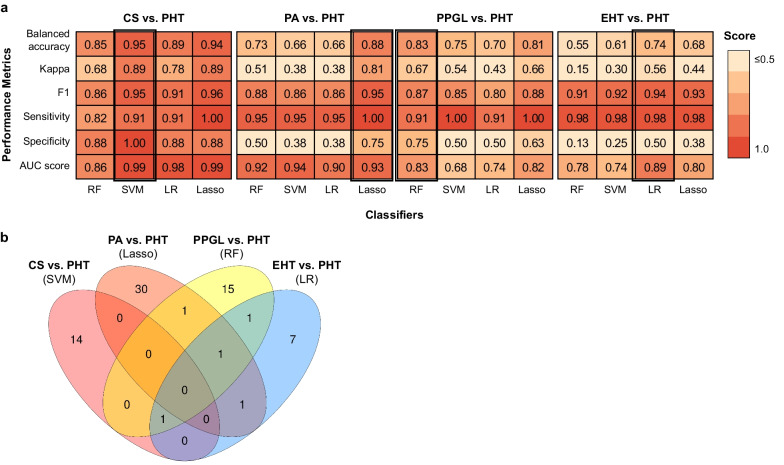
Prediction of endocrine hypertension by blood methylome marks. **a** Heatmap of prediction scores in validation cohorts for each type of endocrine hypertension (CS, PA, PPGL, EHT) versus primary hypertension (PHT), obtained with different machine learning approaches. *RF* random forests, *SVM* support vector machine, *LR* logistic regression, *Lasso* Least Absolute Shrinkage and Selection Operator. **b** Venn diagram of top performing features selected for each comparison (prediction score of reference: balanced accuracy)

Four different machine learning methods—Lasso, Logistic Regression, Random Forest, Support Vector Machine—were used to build a prediction model for each type of endocrine hypertension on subsets of samples (training cohorts), and subsequently tested on remaining samples (validation cohorts). The prediction performance was better for individual types of EHT (CS, PA and PPGL) than for predicting EHT as a whole. Indeed, balanced accuracy against PHT reached 0.95 for CS using SVM, 0.88 for PA using Lasso, 0.83 for PPGL using RF, and 0.74 for EHT using LR (Fig. 3a; selected CpG sites in Additional file 1: Table S4). Misclassified samples (false negatives and false positives) mostly showed a positioning on the global methylome space in regions of overlapping between different hypertension types (Additional file 1: Fig. S2). In the two comparisons with sample numbers imbalance (PA vs. PTH and EHT vs. PHT), down- and up-sampling were used to further explore prediction score, with no major impact on performance (Additional file 1: Fig. S3). The selected sets of CpG sites were specific for each endocrine hypertension type (Fig. 3b). These specific CpG sites, except for just one CpG site in CS, were all present among the differentially methylated CpG sites selected for each type of endocrine hypertension (Additional file 2: Table S1).

## Discussion

In this work, we were able to identify a blood methylome signature discriminating different types of EHT -including CS, PA and PPGL- from PHT. These different conditions are currently diagnosed with specific hormone measurements, representing the gold standard [9]. However, hormone assays are not always conclusive, with accuracies far from perfect [24–26], and quite expensive. This leads to screening strategies that target hypertensive patients with the highest risk of endocrine etiology [3, 4]. These probabilistic approaches could miss cases compared to a full screening of the entire hypertensive population [27]. Specific non-hormonal biomarkers could potentially help in situations where (i) hormone levels are not applicable—for instance, exogenous glucocorticoid administration; (ii) hormone dosages are not informative, due to situations interfering with hormone assays [28]; (iii) hormone dosages are borderline, not providing a proper classification of patients. Some non-hormonal markers of endocrine hypertension have been proposed, including specifically targeted metabolomics [29], and miRNAs [30, 31]. Blood methylation marks may also be suitable for direct measurement of hormone excess on peripheral tissues. Indeed, DNA methylation is dynamic but stably preserved [14] and could potentially reflect the individual impact of hormone excess over time better than hormonal evaluation, that need to be standardized for time, feeding-status, salt and fluid intake, and co-medication, among others.

Beyond the specific benefits for diagnosis, massive molecular screenings in common diseases like hypertension are expected to improve personalized medicine, through the identification of features predicting response to treatment, or specific complications. Addressing these aims will require specific study designs, focused on follow-up. For these up-coming studies, robustness and statistical power will improve by restricting the number of candidate features to be tested. This prior feature selection will be helped by this work and others, providing lists of CpG sites associated with each type of hypertension or with each type of hypertensive treatment [32].

Of note, the methylome signatures identified here are impacted by white blood cell count. Indeed, neutrophils increase and lymphocytes decrease in case of CS and, to a lesser extent, of PA [33–36]. However, we showed that white blood cell count was contributing to a limited part of methylome variability. To consider this effect, the methylome signatures provided here were adjusted for white blood cell count. The proportion of neutrophils was used as a unique proxy for this adjustment, given the high correlation with proportions of lymphocytes and lymphocyte subtypes.

Here, we identified blood DNA methylation loci associated with EHT. The blood DNA methylome profile associated with the different types of hormonal excess was also explored, with the identification of genes differentially methylated, such as *FKBP5* in CS and *CD300LG* in PA. *FKBP5* encodes a co-chaperone regulating the glucocorticoid receptor activity [37], and its increased expression and decreased methylation in blood have already been described in CS [18, 38]. Moreover, stress-induced epigenetic up-regulation of *FKBP5* has been associated with an increased risk of cardiovascular events [39]. *CD300LG* encodes a vascular endothelial cell adhesion molecule, which is implicated in leukocyte binding and transmigration, and whose expression seems to be differently influenced by the local environment in different tissues [40]. Aldosterone is known to induce endothelial dysfunction [41] and to promote a pro-inflammatory phenotype, with vascular infiltration of monocytes, macrophages, and lymphocytes, as demonstrated in both animal models of hypertension [42, 43] and human vascular endothelial cells [44]. Whether *CD300LG* possibly contributes to the effect of aldosterone on vascular endothelium and lymphocyte migration, and whether this association represents a specific trait of aldosterone-induced hypertension, remains to be established. One study explored C*D300LG* variants in a mouse model of hypertension, but with uncertain conclusions [45]. For a majority of other genes, the biological relevance in endocrine hypertension remains to be explored, including the impact of DNA methylation of gene expression.

The hypothesis of a common blood methylome signature of EHT was also addressed. When we compared CS-, PA- and PPGL-related signatures, a limited number of methylation marks were shared, suggesting the prominence of individual endocrine hypertension signatures over a common signature of EHT. However, sample projections based on the variability of methylation levels showed overlap between PA and PPGL, and a tendency of CS to cluster separately. Indeed, secondary aldosteronism is common in PPGL [46] and could in part explain this similarity with PA. To which extent an endocrine-related molecular signature of hypertension shared between CS, PA and PPGL, but distinct from PHT, is induced remains to be determined. By directly comparing EHT as a whole to PHT, we provide here a set of methylome marks, which may facilitate the identification of such molecular signatures.

Whether these methylation marks better reflect the risk of complications related to hormone excess also remains to be elucidated. Of note, the methylation signatures identified here were based on whole methylome analysis. Routine use of such a tool would be limited by cost and complexity. Reliable biomarkers should allow the prediction of a clinical endpoint of interest more easily, at a lower cost and over a shorter time span than the direct measurement of the clinical end point [47]. For methylation marks, this would imply a technology transfer to targeted measurements, such as pyrosequencing, methylation specific-MLPA, methylation specific-high resolution melting analysis, or nanopore sequencing [48, 49]. Setup and validation of suitable targeted assays have to be performed for the identified methylome marks of EHT.

A potential interest of selected methylome marks could be the integration in a targeted multi-omic chip for the diagnosis of endocrine hypertension (ENSAT-HT project; http://www.ensat-ht.eu). Whether these methylation marks could positively impact the prediction of a multi-modal classifier remains to be determined, especially for sensitive detection of the rarest forms of endocrine hypertension, namely CS and PPGL.

This work aimed to develop machine learning models with methylome data to predict endocrine hypertension. This approach might be seen as too simple regarding the pathophysiological complexity of these conditions, and the extensive and complex diagnosis workup currently needed to call these conditions. However, machine learning could help in leading a step forward toward a robust and accurate screening tool and could provide a unique opportunity to understand the complex relationships within an omic dataset and a complex phenotype. On the other side, this data-driven strategy may be impacted by biases, including recruitment biases—in expert centers and optimal conditions-, and representation biases—considering the rare prevalence of CS or PPGL compared to the other types of hypertension. This latter risk is mitigated here by the study design splitting samples into independent training and validation cohorts. Finally, the heterogeneity of sample sizes for the different types of endocrine hypertension may impact the machine learning process. However, the group of EHT was balanced in terms of endocrine hypertension types when analyzed as a whole, and optimized strategies were used for unbalanced comparisons, including balanced accuracy measurement, or down- and up-sampling of the training cohorts.

## Conclusions

Endocrine hypertension can be identified by blood DNA methylation markers, with specific methylation signatures for each type of endocrine disorder.

## Methods

### Patients and samples

A total of 255 blood samples were collected in eight different centers, as part of the European ENSAT-HT study (http://www.ensat-ht.eu). The cohort included patients with PHT (*n* = 42) and patients with different types of EHT, namely CS (*n* = 57), PA (*n* = 101), or PPGL (*n* = 55) (Additional file 5: Table S5). The diagnosis was made according to the current guidelines for screening and management of each specific disease [4, 50–52]. Diagnosis of PHT also required the exclusion of endocrine hypertension and other secondary causes (renal disease, pharmacological cause and obstructive sleep apnea syndrome) as well as the exclusion of patients with low-renin hypertension. Patients with uncertain diagnosis, those with pregnancy, severe comorbidities (including heart failure, chronic kidney disease, active malignancy) were also excluded.

### Whole-genome DNA methylation measurement

Leukocyte DNA was extracted from EDTA blood samples, using the DNA Isolation kit for Mammalian Blood (Roche, Basil, Switzerland). DNA quality was assessed on a Genomic DNA ScreenTape system (Agilent, Santa Clara, CA, US) and quantified using a Qubit 3.0 Fluorometer (Thermofisher, Waltham, MA, US). DNA was treated by bisulfite and then hybridized to the Infinium MethylationEPIC BeadChip (Illumina, San Diego, CA, US; ~ 865,000 sites), starting from 500 ng of DNA. All experiments were performed following the manufacturer’s instructions at the P3S Post-Genomic Platform of Sorbonne University (Paris, France).

### Bioinformatics and statistics

All samples passed the quality controls provided by the Genome Studio software (v. 2011.1; Illumina). Data were exported in Intensity Data (IDAT) format and then processed using the *minfi* package (v. 1.32.0) [53] in the R software environment (v. 3.6.3) (https://cran.r-project.org/). An additional quality control of samples was performed based on probe detection quality, confirming the good quality of each sample (mean detection *p* value < 0.05).

Data were normalized using the stratified quantile normalization procedure implemented in the “preprocessQuantile” *minfi* function [54] and the methylation score for each CpG probe was extracted as a β-value. Then, the *ChAMP* package (v. 2.16.1) was used to filter the probes [55]. A total of 731,635 CpG sites passed the following criteria: detection *p* value < 0.01, presence of the targeted CpG site, absence of frequent SNPs in the probe, single hybridization hit, autosomal target. Of note, filtering the probes before normalization versus after normalization did not significantly impact the values (data not shown).

The significant components of variation in the dataset were assessed using the singular value decomposition method (SVD) for methylation data [56], and a detected batch effect (Slide) was corrected using the “ComBat” method [57], as implemented in the *ChAMP* package.

White blood cell count of subpopulations (neutrophils, lymphocytes B, lymphocytes T4, lymphocytes T8, lymphocytes NK, monocytes) were estimated by the reference-based “RefbaseEWAS” method [58] implemented in the *ChAMP* package (Additional file 6: Table S6). Since neutrophils were the most represented cell type in all samples, and since the proportions of neutrophils and lymphocytes were anti-correlated (Pearson’s *r* = − 0.96; Additional file 1: Fig. S1c, d), the estimated proportion of neutrophils was chosen as the unique proxy reflecting variations in white blood cell count.

M-values, used for statistical analysis, were calculated from β-values (log2 ratio of the intensities of methylated vs. unmethylated probes) using the *lumi* package (v. 2.36.0) [59].

The global data structure was assessed on β-values by principal component analysis (PCA), using all the CpG probes. The most variable CpG probes were selected based on their Standard Deviation (SD cutoff: 0.4, *n* = 48,452 probes) among all samples. Differentially methylated CpG sites were identified using the *Limma* package (v. 3.40.6) [60] for each of the following comparison: EHT vs. PHT, CS vs. PHT, PA vs. PHT, PPGL vs. PHT. For the EHT vs. PHT comparison, the EHT group was obtained by a random sampling of CS, PA and PPGL (*n* = 55 samples for each type of endocrine hypertension), to avoid imbalance between the endocrine hypertension types. The estimated neutrophil count and age were included as covariates and CpG sites were selected based on an adjusted *p* value < 0.05 (Benjamin-Hochberg correction procedure; Additional file 2: Table S1). Gene set enrichment analysis of genes associated with differentially methylated CpG sites was performed using the “gometh” method (KEGG chosen as collection of pathways to test) implemented in the *missmethyl* package (v. 1.18.0) [61], adjusting for the number of CpG sites associated to each gene [62] (Additional file 3: Table S2). For each endocrine hypertension type, differentially methylated genes were selected when they presented at least three differentially methylated CpG sites in each comparison and a − log10(*p* value) > 5 (Additional file 4: Table S3).

For predicting endocrine hypertension, the same four comparisons described above were considered for supervised learning (CS vs. PHT, PA vs. PHT, PPGL vs. PHT, and EHT vs. PHT). For each comparison, training (80%) and validation (20%) samples were randomly selected by maintaining the initial proportion of samples in each hypertension type in each comparison (Additional file 1: Table S7). Starting from the most variable CpG sites previously selected (*n* = 48,452 probes with M-value SD > 0.4), the most discriminating CpG sites for each comparison (*n* = 200 for CS vs. PHT, *n* = 141 for PA vs. PHT, *n* = 70 for PPGL vs. PHT, and *n* = 135 for EHT vs. PHT) were pre-selected from the training sets using the *boruta* package (v. 0.3) [63] (Additional file 1: Table S8). For each comparison, four different classifier methods were then used: (i) penalized Lasso (Least Absolute Shrinkage and Selection Operator) regression with tenfold cross-validation, using the *glmnet* package (v. 4.0–2) [64]; (ii) Support Vector Machine (SVM) [65]; iii) Random Forest (RF) [66]; iv) Logistic Regression (LR) [67] with Fast Correlation-Based Filter (FCBF) [68] using the *orange* toolbox (v. 3.30.1) [69] (Additional file 1: Table S9). The trained models were tested on the validation sets for each comparison, and the prediction performance was evaluated using balanced accuracy, sensitivity, specificity, F1, Kappa, and AUC scores. The model training was further evaluated using up-sampling and down-sampling (70).

Group comparisons were performed using Student’s t test for variables normally distributed, or chi-square test for binary categorical variables. All tests were computed in the R software environment.

## Supplementary Information

**Additional file 1: Fig. S1.** Components of variation in the whole methylome dataset. a) Scree plot representing the percentage of explained variability by the first five principal components of PCA performed on the whole dataset (n=731,635 CpG sites, n=255 samples). b) Singular value decomposition (SVD) plot assessing the correlation between the first five significant components of variation in the dataset and other biological factors of interest (Hypertension type –PHT, CS, PA, PPGL-, age, sex, cell composition–neutrophils used as the unique proxy). c) Correlation between the proportion of neutrophils and of lymphocytes. d) Correlation plot between the proportion of neutrophils and of the other different cell subtypes. **Fig. S2.** Misclassified sample positioning in the global structure of blood DNA methylation. Samples with discrepant methylome prediction and hormonal status are indicated by the white squares. **Fig. S3.** Heatmap of prediction scores in validation cohorts for primary aldosteronism (PA) and endocrine hypertension (EHT) versus primary hypertension (PHT), obtained with different machine learning approaches, after up-sampling and down-sampling the training cohort. *RF* random forests, *SVM* support vector machine; *LR* logistic regression; *Lasso* Least Absolute Shrinkage and Selection Operator. **Table S4.** Top performing methylome features. List of CpG sites selected for predicting each type of endocrine hypertension, based on the best performing method for each comparison. **Table S7.** Training/Validation split of samples. **Table S8.** Parameters chosen for Boruta package. For details, see https://github.com/scikit-learn-contrib/boruta_py. **Table S9.** Parameters chosen for different machine learning models for classification task.

**Additional file 2: Table S1.** Differentially methylated CpG sites in endocrine hypertension.

**Additional file 3: Table S2.** Enriched signaling pathways in endocrine hypertension.

**Additional file 4: Table S3.** Significant differentially methylated genes in endocrine hypertension.

**Additional file 5: Table S5.** Sample characteristics.

**Additional file 6: Table S6.** Methylome-based estimation of blood cell composition.

## Data Availability

The methylome dataset generated and analyzed during the current study is available in the EMBL-EBI BioStudies repository (Reference Number: S-BSST831).
