# Supplementary material for: Whole blood methylome-derived features to discriminate endocrine hypertension
Source: Clin Epigenetics. 2022 Nov 3;14:142. doi: 10.1186/s13148-022-01347-y (PMC9635165; doi:10.1186/s13148-022-01347-y)
Supplement: Supplementary file 1 — Additional file 1: Fig. S1. Components of variation in the whole methylome dataset. a) Scree plot representing the percentage of explained variability by the first five principal components of PCA performed on the whole dataset (n=731,635 CpG sites, n=255 samples). b) Singular value decomposition (SVD) plot assessing the correlation between the first five significant components of variation in the dataset and other biological factors of interest (Hypertension type –PHT, CS, PA, PPGL-, age, sex, cell composition–neutrophils used as the unique proxy). c) Correlation between the proportion of neutrophils and of lymphocytes. d) Correlation plot between the proportion of neutrophils and of the other different cell subtypes. Fig. S2. Misclassified sample positioning in the global structure of blood DNA methylation. Samples with discrepant methylome prediction and hormonal status are indicated by the white squares. Fig. S3. Heatmap of prediction scores in validation cohorts for primary aldosteronism (PA) and endocrine hypertension (EHT) versus primary hypertension (PHT), obtained with different machine learning approaches, after up-sampling and down-sampling the training cohort. RF random forests, SVM support vector machine; LR logistic regression; Lasso Least Absolute Shrinkage and Selection Operator. Table S4. Top performing methylome features. List of CpG sites selected for predicting each type of endocrine hypertension, based on the best performing method for each comparison. Table S7. Training/Validation split of samples. Table S8. Parameters chosen for Boruta package. For details, see https://github.com/scikit-learn-contrib/boruta_py. Table S9. Parameters chosen for different machine learning models for classification task. [file 13148_2022_1347_MOESM1_ESM.docx]

# Supplementary material

## Supplementary Figures


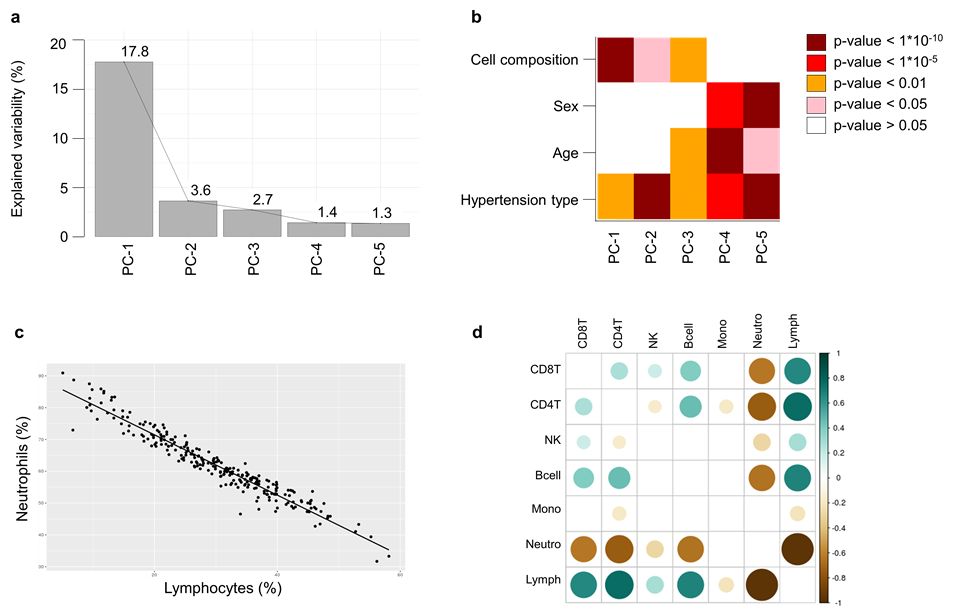


**Supplementary Figure 1. Components of variation in the whole methylome dataset.** a) Scree plot representing the percentage of explained variability by the first five principal components of PCA performed on the whole dataset (n=731,635 CpG sites, n=255 samples). b) Singular value decomposition (SVD) plot assessing the correlation between the first five significant components of variation in the dataset and other biological factors of interest (Hypertension type –PHT, CS, PA, PPGL-, age, sex, cell composition–neutrophils used as the unique proxy). c) Correlation between the proportion of neutrophils and of lymphocytes. d) Correlation plot between the proportion of neutrophils and of the other different cell subtypes.


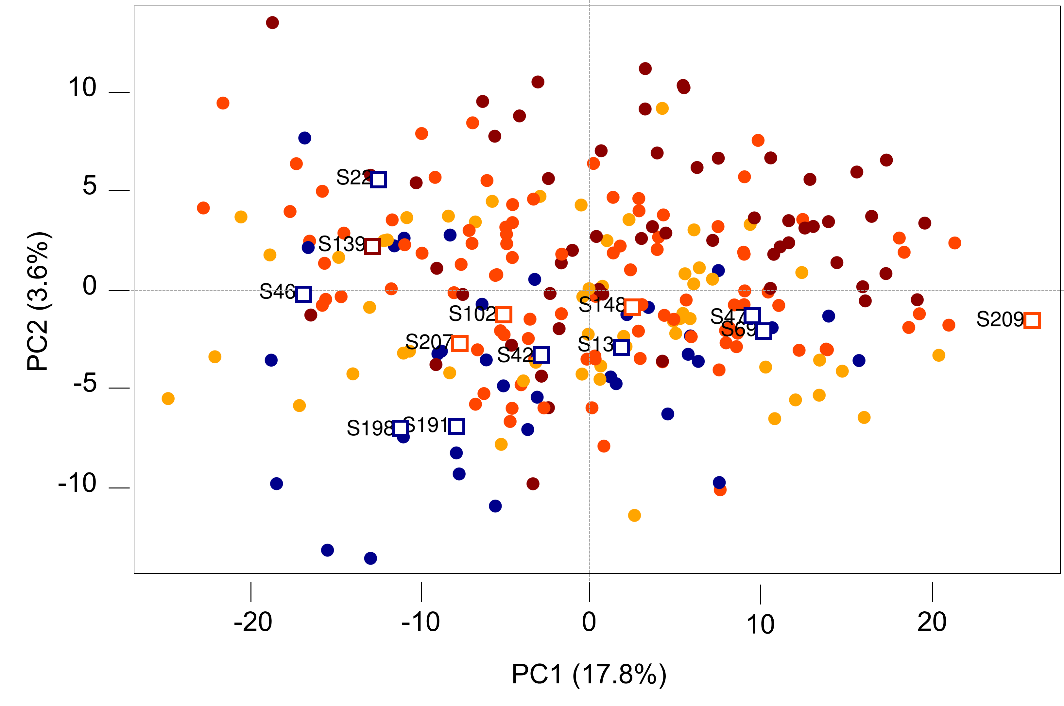


**Supplementary Figure 2. Misclassified sample positioning in the global structure of blood DNA methylation.** Samples with discrepant methylome prediction and hormonal status are indicated by the white squares.

**
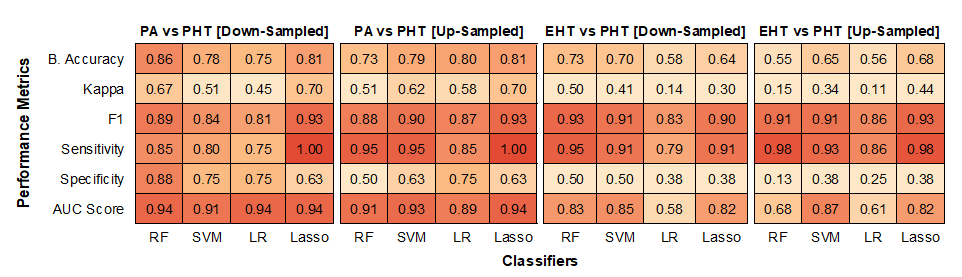
**

**Supplementary Figure 3**. Heatmap of prediction scores in validation cohorts for primary aldosteronism (PA) and endocrine hypertension (EHT) versus primary hypertension (PHT), obtained with different machine-learning approaches, after up-sampling and down-sampling the training cohort. RF: random forests, SVM: support vector machine; LR: logistic regression; Lasso: Least Absolute Shrinkage and Selection Operator.

## Supplementary Tables

**Supplementary Table 1 - Differentially methylated CpG sites in endocrine hypertension**

“Supplementary_Table1.xlsx” file

**Supplementary Table 2 – Enriched signaling pathways in endocrine hypertension.**

“Supplementary_Table2.xlsx” file

**Supplementary Table 3 - Significant differentially methylated genes in endocrine hypertension**

“Supplementary_Table3.xlsx” file

| **CS vs PHT**  ***SVM*** |  | **PA vs PHT**  ***LASSO*** |  | **PPGL vs PHT**  ***RF*** |  | **EHT vs PHT**  ***LR*** |  |
| --- | --- | --- | --- | --- | --- | --- | --- |
| CpG site | Gene | CpG site | Gene | CpG site | Gene | CpG site | Gene |
| cg24166814 |  | cg02016407 |  | cg13741289 | LAP3 | cg11334870 | STK10 |
| cg05158757 |  | cg07588263 | C8orf40 | cg08458738 |  | cg25901000 | SLC39A11 |
| cg03546163 | FKBP5 | cg04751035 |  | cg04318704 | ZNF702P | cg23792383 | KLF5 |
| cg07226162 |  | cg07240190 | OSTCP1 | cg24898815 |  | cg02384475 |  |
| cg25597390 |  | cg18806716 | MAP3K8 | cg02635865 | RAB22A | cg13188409 | SNX14 |
| cg05468843 | IL10RA | cg00134134 | RALGPS2 | cg23236554 | FSTL4 | cg09766769 | ROGDI |
| cg04895321 | NMT2 | cg15255086 |  | cg26121782 | LMO1 | cg23356920 | EIF4G1 |
| cg24053343 | ANKS1A | cg13250850 | RABGGTB | cg07708653 | HPDL | cg07219314 | ZDHHC8P |
| cg17788362 | SYNCRIP | cg07222309 |  | cg10493027 | TANK | cg10864924 | RAB11FIP2 |
| cg16478871 | XPO6 | cg25901000 | SLC39A11 | cg23379342 | TAMM41 | cg00276040 | LOC101929431 |
| cg27107150 | TPSG1 | cg17211447 | APITD1 | cg13354988 | ACP1;ACP1 | cg21839504 | STK10 |
| cg12943692 |  | cg22092780 | LRP11 | cg23108367 | CPD |  |  |
| cg27064709 | CDC42BPA | cg16304914 | ST3GAL3 | cg14943877 | DUSP5 |  |  |
| cg11605358 |  | cg05850327 | GJB2 | cg00276040 | LOC101929431 |  |  |
| cg00276040 | LOC101929431 | cg24140629 | SYPL1 | cg22370326 | ANKRD57 |  |  |
|  |  | cg23304085 | CDKL3 | cg16503386 |  |  |  |
|  |  | cg18675097 | NKAPL | cg16564842 | SNX10 |  |  |
|  |  | cg16311044 | KIAA1875 | cg25901000 | SLC39A11 |  |  |
|  |  | cg10726445 | LMO7 | cg10864924 | RAB11FIP2 |  |  |
|  |  | cg10493027 | TANK |  |  |  |  |
|  |  | cg10358780 | GOLT1B |  |  |  |  |
|  |  | cg22111009 | C5orf47 |  |  |  |  |
|  |  | cg19696333 | IKZF5 |  |  |  |  |
|  |  | cg13188409 | SNX14 |  |  |  |  |
|  |  | cg14434028 | TIA1 |  |  |  |  |
|  |  | cg20850918 | WEE1 |  |  |  |  |
|  |  | cg17880919 | LINC01192 |  |  |  |  |
|  |  | cg16415999 | YTHDF3 |  |  |  |  |
|  |  | cg17518036 | PFDN6 |  |  |  |  |
|  |  | cg26026297 | ATP8A1 |  |  |  |  |
|  |  | cg26268145 | PDE2A |  |  |  |  |
|  |  | cg14784953 |  |  |  |  |  |
|  |  | cg04542433 | BEAN |  |  |  |  |

**Supplementary Table 4 – Top performing methylome features.** List of CpG sites selected for predicting each type of endocrine hypertension, based on the best performing method for each comparison.

**Supplementary Table 5 – Sample characteristics**

“Supplementary_Table5.xlsx” file

**Supplementary Table 6 – Methylome-based estimation of blood cell composition**

“Supplementary_Table6.xlsx” file

| **Comparison** | **CS vs PHT** | | | **PA vs PHT** | | | **PPGL vs PHT** | | | **EHT vs PHT** | | |
| --- | --- | --- | --- | --- | --- | --- | --- | --- | --- | --- | --- | --- |
| Samples (n) | CS | PHT | Total | PA | PHT | Total | PPGL | PHT | Total | EHT | PHT | Total |
| *Training Cohort* | 46 | 34 | 80 | 81 | 34 | 115 | 44 | 34 | 78 | 170 | 34 | 204 |
| *Validation Cohort* | 11 | 8 | 19 | 20 | 8 | 28 | 11 | 8 | 19 | 43 | 8 | 51 |
| *Total* | *57* | *42* | *97* | *101* | *42* | *143* | *55* | *42* | *97* | *213* | *42* | *255* |

**Supplementary Table 7 – Training/Validation split of samples**

| **Parameter** | **Chosen value** |
| --- | --- |
| Estimator | RandomForestClassifier(n_jobs=-1,  class_weight='balanced',  max_depth=5,  bootstrap=True,  criterion='gini') |
| n_estimator | ‘auto’ |
| max_iter | 5000 |
| Verbose | 2 |
| random_state | 0 |

**Supplementary Table 8 - Parameters chosen for Boruta package.** For details, see <https://github.com/scikit-learn-contrib/boruta_py>.

| **Model** | **Parameters** | **Chosen value** |
| --- | --- | --- |
| SVM | Cost (C) | 1.0 |
| SVM | Regression loss epsilon (ε) | 0.1 |
| SVM | Kernel | RBF, exp(-auto\|x-y\|^2^) |
| SVM | Numerical tolerance | 0.001 |
| SVM | Iteration limit | 100 |
| LR | Regularisation type | Ridge (L2) |
| LR | Cost strength (C) | 1.0 |
| RF | Num of trees | 10 |
| RF | Max num of considered features | Unlimited |
| RF | Replicate training | No |
| RF | Maximal tree depth | Unlimited |
| RF | Stop splitting nodes with maximum instances | 5 |

**Supplementary Table 9 - Parameters chosen for different machine learning models for classification task.**
